# Signs of the Times: Biomarkers in Perspective

**DOI:** 10.1289/ehp.114-a700

**Published:** 2006-12

**Authors:** Charles W. Schmidt

During the last decade, the health sciences have experienced a major shift in orientation. With the rise of genomics and advances in molecular biology, scientists have increasingly moved away from population-based approaches to health toward studies of disease susceptibility among individuals. That change is reflected by the push for personalized medicine, which targets the underlying susceptibilities that make some people prone to particular diseases. It’s also reflected by recent goals for personalized exposure assessment in environmental health, and efforts to understand why some people seem particularly vulnerable to the harmful effects of pollution and other environmental toxicants.

The focus on individuals could make public health strategies more effective by allowing practitioners to direct resources toward those with the greatest need. But the success of these efforts will depend largely on the continued identification of biomarkers that reflect the individual’s health status and risk at key time points.

Scientists rely on biomarkers to track each phase of the dose–response continuum, from exposure through effect. In a broad sense, biomarkers include physical parameters that can be clearly anchored to a disease or class of diseases. In medicine, they can include measures such as heart rate or serum cholesterol, both of which correlate directly with cardiac disease risk. For environmental health purposes, biomarkers include a range of additional exposure-related indices, such as pollutant measures in tissues and bodily fluids; exposure-induced changes in cells, proteins, DNA, and other molecules; and inherited gene variations that influence how individuals respond to their environments. Single-nucleotide polymorphisms (SNPs), for instance, which are simple inherited gene variations, can increase or lessen disease susceptibility following environmental exposures.

Biomarker investigations are now an integral part of environmental health research. Through its Exposure Biology Program, established in 2006, the NIEHS will commit substantial resources to the search for biomarkers, focusing particularly on those that reflect the human response to environmental agents. Likewise, the EPA Office of Research and Development has committed at least $3 million annually since 2000 to biomarker investigations.

But even as the public health community ramps up its efforts in this area, the search for new biomarkers has been slow and often frustrating. Scientists can propose biomarkers on the basis of animal research or limited studies in humans, but to confirm their relevance to broad human populations, biomarkers must be validated in population-based studies often involving large cohorts, ideally using prospective studies that involve repeated sampling of individuals.

Repeated sampling assures scientists that biomarkers reflect actual disease processes, instead of measurement errors or other incidental variations. Yet prospective studies that incorporate multiple sampling rounds for biomarker validation are time-consuming and expensive, so very few have been conducted, says John Groopman, chairman of the Department of Environmental Health Sciences at the Johns Hopkins Bloomberg School of Public Health. “That really hinders our ability to make intermediate linkages between exposure and disease outcome,” he explains. Consequently, biomarkers that reflect the full response spectrum from exposure through effect have been identified for just a few agents, notable among them benzene, aflatoxin B_1_, and UV radiation.

## The Basis of the Field

The use of biomarkers in the environmental health sciences arguably dates back to the early 1970s, with Herbert Needleman’s groundbreaking work on lead neurotoxicity in children. Now a professor at the University of Pittsburgh Medical Center, Needleman was an assistant professor at Harvard Medical School when he used blood lead as a biomarker to show that even at the lowest detectable levels, lead could impair a child’s IQ. With that finding, he conclusively linked a lead biomarker to cognitive decline and sparked efforts to remove the metal from gasoline, which was the largest source of human exposure at the time.

But though Needleman was using blood lead as a biomarker, he didn’t articulate it as such. The terminology used in biomarker research today didn’t actually emerge until the early 1980s. During that time, environmental health sciences professor Frederica Perera and medical professor I. Bernard Weinstein, both of the Columbia University Mailman School of Public Health, proposed molecular epidemiology—the use of biomarkers to link human environmental exposures with illness—as a new approach to the study of cancer. They also proposed four categories of biomarkers: internal dose, biologically effective dose, early biologic response, and susceptibility. They suggested that exposure assessment should turn its focus from population-based estimates of dose to the quantification among individuals of biologically effective dose, defined as the amount of a chemical interacting with critical cellular targets.

In a paper published in volume 3, issue 12 (1982) of *Carcinogenesis* with colleagues from the National Cancer Institute (NCI), Perera and Weinstein reported that they had detected cellular adducts of DNA and the carcinogen benzo[*a*]pyrene in lung tissue, and in several cases blood cells. In that particular case, the adduct was the biomarker, and the critical cellular target was DNA. But the numbers of patients tested in the study was too small to draw definitive conclusions about risk.

Even so, the concept of a biologically effective dose survived and flourished in the scientific literature. Scientists acknowledged that to fully understand toxic responses, they would have to identify key events along the pathway from exposure to disease. But their early efforts to do so were limited in part by the lack of an organized framework for pursuing biomarker studies, says Nathaniel Rothman, a senior investigator in the NCI’s Occupational and Environmental Epidemiology Branch.

“[Scientists] would try to communicate with each other, but sometimes it would be like ships passing in the night,” he says. “One would be talking about using an exposure biomarker to ask one type of research question, and another about applying an early biologic effect marker to ask a different research question, and neither would realize they weren’t talking about quite the same thing.”

In 1987, the National Research Council convened a committee to investigate how biomarkers were being developed and used in the environmental health sciences. The conclusions were documented in a seminal paper published in the October 1987 issue of *EHP*, which described the four basic biomarker groupings still in use today: exposure biomarkers (which include markers of external exposure and of internal dose); biomarkers of biologically effective dose; effect biomarkers (which include markers of health impairment or recognized disease, early disease precursors, or peripheral events that predict health impairment); and susceptibility biomarkers (which include intrinsic genetic or other characteristics or preexisting diseases that result in an increase in internal dose, biologically effective dose, or target tissue response).

The 1987 paper also introduced the concept of a “continuum of biological events” in toxicity, and proposed that biomarkers could be used to delineate each event within the continuum, from exposure, to internalized dose, to biologically effective dose, to altered molecular structure, and finally to clinical disease.

“These articles really made a critical contribution,” Rothman says. “They helped codify terms and gave everyone a flexible structure to work with.”

## Adductive Reasoning

During the next decade, biomarker research took hold and produced some important success stories, particularly in the area of exposure biomarkers. The emphasis on exposure was driven by growing excitement over the adducts that some chemicals form with proteins and DNA. As biomarkers, adducts serve a number of key purposes: they reflect individual exposures, and they also focus exposures on key toxicological targets within the cell. What’s more, adducts can provide evidence for metabolic steps in the exposure–response continuum. That’s because some compounds can bind with proteins or DNA only after being metabolically transformed.

Groopman was one of the first to conclusively link DNA adducts with disease, specifically human liver cancer induced by aflatoxin B_1_, a carcinogenic product of a mold found on peanuts and other grains. Using experimental animals, he started by showing that aflatoxin exposure was reflected in DNA–aflatoxin adducts in urine. He then demonstrated that the biomarkers could be detected in human urine samples obtained from exposed populations in Asia. Equally significant, he found the adduct concentrations rose in proportion to dose, thus confirming their value as quantifiable indicators of personal exposure. Finally, in a classic nested case–control study carried out in a cohort in Shanghai and described in the September 1994 issue of *Cancer Epidemiology Biomarkers & Prevention*, he showed that a urinary DNA–aflatoxin adduct was more strongly associated with risk of liver cancer than urinary aflatoxin metabolites.

“This was significant, because Groopman showed that DNA adducts reflected more than just aflatoxin exposure,” says Rothman. “He also showed that people with liver cancer, given detectable levels of any measured aflatoxin metabolite, had a tendency to produce more DNA adducts, which suggests that this biomarker incorporates other meaningful information.” Further, Groopman and his colleagues showed that biomarkers of aflatoxin exposure interacted with hepatitis B infection to produce very high risks of liver cancer.

One of the consequences of unrepaired DNA–aflatoxin adducts is mutation in cancer-related genes. In the early 1990s, NCI Laboratory of Human Carcinogenesis chief Curtis Harris, Mehmet Ozturk of Harvard Medical School, and their colleagues discovered a specific aflatoxin-related mutation in the *p53* tumor suppressor gene in Chinese and African populations occurring in liver cancer from people exposed to aflatoxin and infected with hepatitis B. This *p53* mutation can be measured in DNA from blood plasma and has recently been shown to be a biomarker of aflatoxin exposure and effect by Ruggero Montesano and colleagues at the International Agency for Research on Cancer.

Meanwhile, other researchers were also making key advances. Lars Ehrenberg, a professor at Stockholm University in Sweden, showed that hemoglobin adducts could accumulate a record of environmental exposure extending over the life of red blood cells (which averages roughly 120 days). Those studies gave rise to the seminal work of Steven Tannenbaum, a professor of toxicology and chemistry at the Massachusetts Institute of Technology, who demonstrated that hemoglobin adducts with aromatic amines could not only provide key exposure biomarkers for these compounds, but could also predict elevated risks for human bladder cancer. Stephen Hecht, a professor of cancer prevention at the University of Minnesota, found that DNA adducts with tobacco-specific nitrosamines were outstanding indicators of environmental tobacco smoke exposure. According to Groopman, Hecht’s work contributed key advances to our understanding of tobacco smoke exposures among both smokers and nonsmokers, and catalyzed the movement to reduce secondhand smoke exposure in the United States. In a related example, published in the September 2001 issue of *Cancer Research*, Perera and colleagues at Harvard University reported that polycyclic aromatic hydrocarbon (PAH)–DNA adducts in white blood cells obtained from smokers enrolled in a prospective cohort study predicted a threefold elevation in risk for subsequent lung cancer.

Among the most comprehensive efforts was work over the past 15 years by Rothman and Martyn Smith, a professor of toxicology at the University of California, Berkeley, along with colleagues from the NCI, the University of North Carolina at Chapel Hill, and the Chinese Academy of Preventive Medicine (now the Chinese Center for Disease Control and Prevention). They described the relationship between external benzene exposure and urinary metabolites with early biological end points, including peripheral blood cell counts, chromosomal aberrations, and more recently, biomarkers identified by proteomic and expression array technologies. This team also identified subgroups of workers who were particularly susceptible to some of these effects because of genetic variations in metabolic and other pathways. And showing how biomarker studies have regulatory implications, they provided evidence of alterations in hematologic end points at benzene exposures of less than 1 ppm, the current OSHA standard. These findings appeared in the 3 December 2004 issue of *Science*.

## Taking a New Tack

Some of these studies are still ongoing today, along with many more not described here. But by the mid-1990s, it was clear that although progress was somewhat steady for exposure biomarkers—particularly for adducts and urinary metabolites—comparable advances were not being made for effects biomarkers or markers of susceptibility. In 1995, members of the Mickey Leland Urban Air Toxics Research Center, a research facility based in Houston, Texas, convened a symposium to evaluate biomarker progress for a range of air pollutants, including aromatics, PAHs, and metals. At the symposium, participants referred to a number of promising exposure biomarkers, mainly adducts and a variety of urinary metabolites. But among the compounds evaluated, a putative effect biomarker was available only for 1,3-butadiene, that marker being an altered white blood cell called the *HPRT* mutant lymphocyte. Even this biomarker, the participants noted, had uncertain value in environmental settings with multiple low-level exposures. As for susceptibility markers, none were noted at all.

Symposium participants felt that several factors constrained efforts to find biomarkers of effects and susceptibility, among them a lack of sufficient human toxicology data, a shortage of epidemiological studies to interpret measurements, uncertain exposure assessments, and high analytical costs. Another problem, experts noted at the time, is that scientists sometimes didn’t give sufficient thought to how the biomarkers they were using fit with their study designs. And that uncertainty led to problems with biomarker selection and interpretation of results.

For instance, while some urinary metabolites may be adequate to monitor recent, short-term exposures, they may not be appropriate to measure longer-term exposures accumulated over weeks, months, or years. Those metabolites, therefore, would be best applied in cross-sectional study designs, in which the main goal is to determine if a particular compound has been absorbed and excreted. These markers’ ability to indicate long-term exposure, particularly for exposures with substantial intraindividual variation, is limited, making them less suitable for use in studies of patients with diseases that have long latencies.

An important step toward identifying key methodologic issues pertaining to biomarker use was made in the text *Molecular Epidemiology: Principles and Practices* by Perera and Paul A. Schulte of the National Institute for Occupational Safety and Health (NIOSH). Following up on this work, Rothman and colleagues at The Johns Hopkins University and NIOSH produced a framework to help scientists select the optimal biomarkers for particular study designs, or if need be, the best type of study design for a given class of biomarkers. Published in the June 1995 issue of *Cancer Epidemiology Biomarkers & Prevention*, the framework appeared in the form of a matrix that scientists could use to match their selections appropriately. That matrix has since become a standard biomarker teaching tool in several settings.

“Our goal was to facilitate more effective discussions and thinking about the use of biomarkers in epidemiological studies,” Rothman says. “I think it helped people appreciate what kind of biomarker will work and when. You really have to consider the advantages and disadvantages of the markers and studies you plan to use.”

## “Omics” and Beyond

As scientists pushed forward on biomarker research during the 1990s, a profound achievement was about to radically alter the biomedical landscape. In June 2000, a public–private partnership of the NIH and Celera Genomics announced they had completed a rough draft of the human genome. With that announcement, biomarker studies entered a whole new era. Enabled by robotic, high-throughput analytical instruments, which allowed them to scan thousands of genes simultaneously, scientists dramatically accelerated their efforts to find new biomarkers.

Today, scientists routinely compare genomic stretches among patients and healthy individuals, searching for gene variations that could provide markers of effect or susceptibility. Those genes, meanwhile, produce proteins that also can serve as biomarkers, and in some cases, as targets for new drugs and other health-protective interventions. Thus, high-throughput “omic” sciences including genomics, proteomics, and metabolomics now drive major efforts in biomarker selection and identification. “And these new biomarkers have the potential to give us a good deal of mechanistic information,” says Harris.

Even so, the dramatic increase in potential biomarkers hasn’t been matched by a rise in useful, validated biomarkers. Scientists still confront important challenges in their efforts to use these indicators in epidemiological investigations. According to Rothman, new biomarkers have to meet the same criteria as those selected in the past: Are they accurate? Do they measure what they purport to measure? Are they stable and reliable in the laboratory? Are they sufficiently sensitive? Are they sufficiently specific to the disease of interest?

The tremendous enthusiasm for SNPs as susceptibility biomarkers has been tempered over time by a lack of successful identifications. John P.A. Ioannidis, a professor at the University of Ioannina School of Medicine in Greece, reports in a 4 October 2006 editorial in the *Journal of the National Cancer Institute* that among 16 SNPs implicated in breast cancer, none were conclusively linked to the disease by scientists participating in a large-scale evaluation. Likewise, Ioannidis adds that meta-analysis of 16 additional candidate gene variants for breast cancer revealed what appeared to be questionable significance with regards to the disease.

Regina Santella, a professor of environmental health sciences at the Mailman School of Public Health, says, “The effects of . . . SNPs are likely to be small. I think it’s going to be really hard to find SNPs and genotypes that we really believe have an impact on health. It’s not going to be one or two of them—it’s going to be multiple genotypes interacting together.”

Echoing that view, Harris suggests a leading edge in research could be directed toward multiple biomarkers—including microRNA, mRNA, protein, and gene expression profiles—linked by integrated models that predict disease risk according to numerous factors. Adding to this, Groopman says that scientists should also consider biomarkers that describe how environmental chemicals interact with infectious agents and bacteria.

“That’s a tremendously exciting opportunity,” Groopman says. “Another area that’s ripe for exploration concerns the examination of protein changes in blood as a consequence of exposure and disease. The ability to explore a well-established protein biomarker repository in blood that we already have now poses a major opportunity.”

Yet even as these opportunities inspire additional research, they also serve as a reminder of how far we still have to go. Biomarkers can reveal key aspects of the body’s response to its environment and perhaps suggest new strategies to protect public health. But to realize those opportunities, society itself must be willing to bear the costs of biomarker validation and the ethical challenges that come with exposing individuals’ vulnerabilities to disease. Assuming those conditions are met, the ambitious goals of personalized medicine, with its commensurate benefits, may one day be reached.

## Figures and Tables

**Figure:**
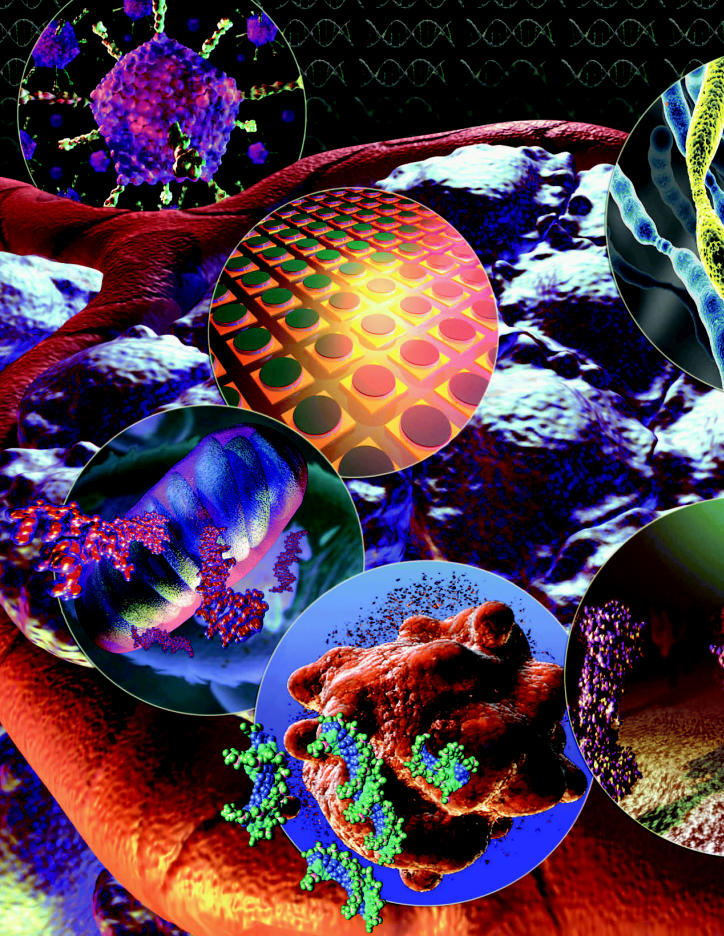


**Figure:**
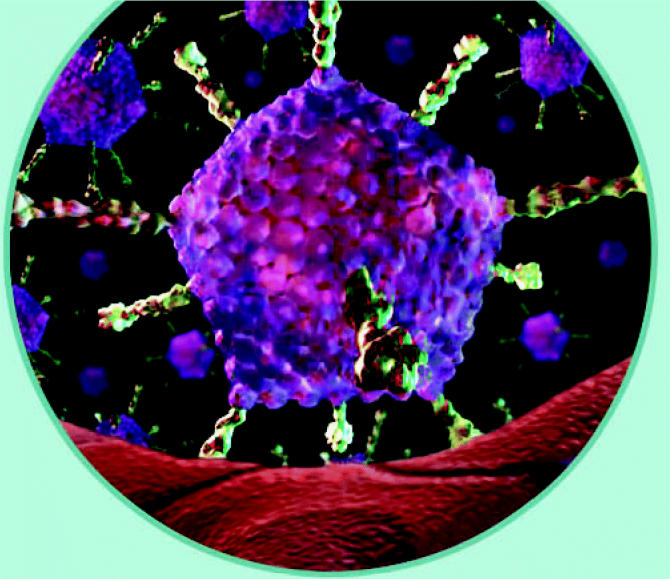
**Exposure biomarkers** estimate the actual internal dose resulting from the exposure, such as bloodborne viral particles.

**Figure:**
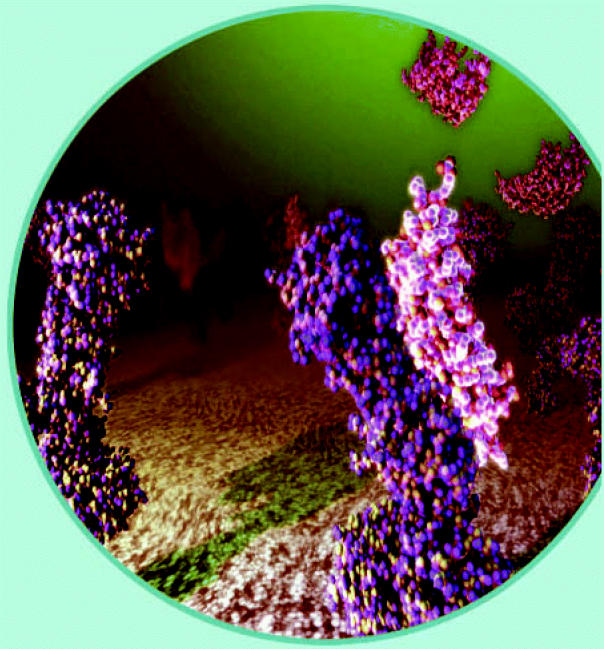
Biomarkers of **biologically effective dose** assess the interaction of toxicants with molecular targets such as protein receptors.

**Figure:**
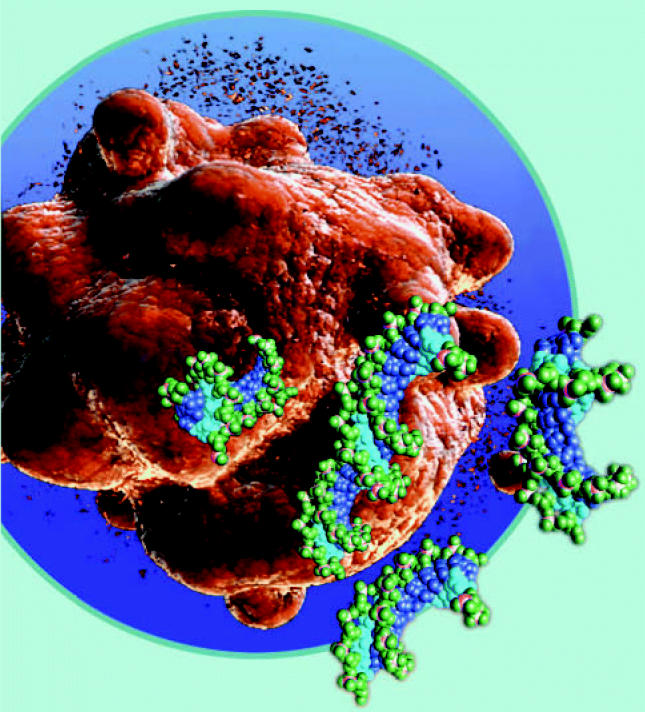
**Effect biomarkers** reflect health impairment or recognized disease, early disease precursors, or peripheral events that predict health impairment, such as apoptosis.

**Figure:**
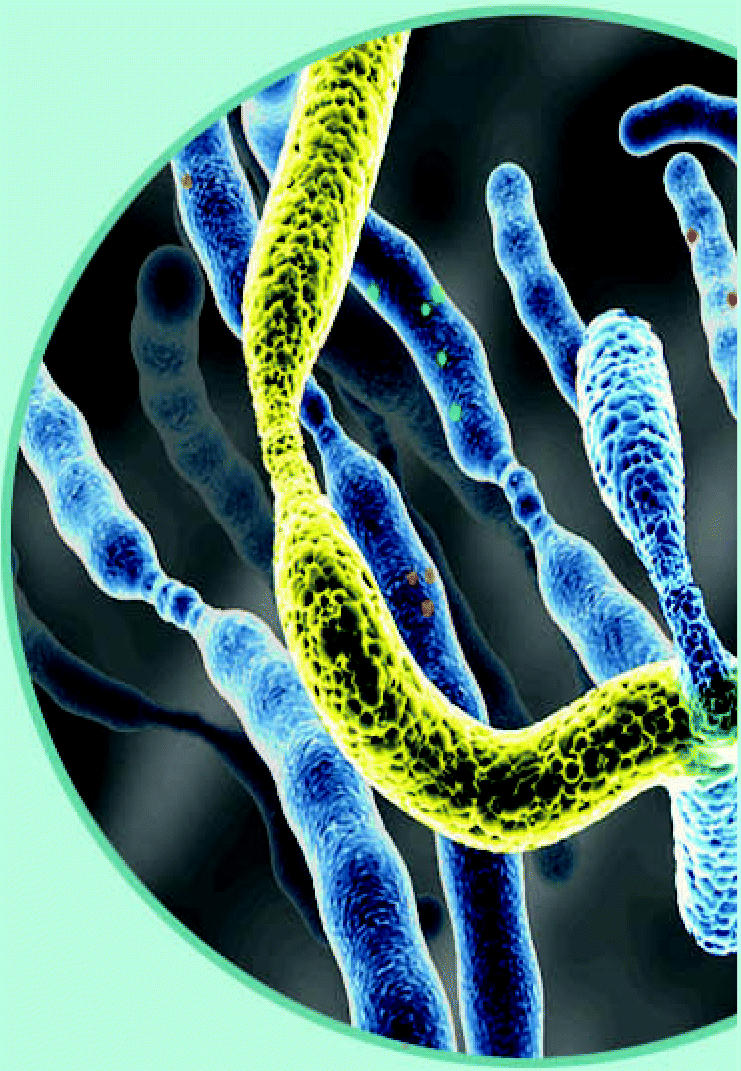
**Susceptibility markers** include intrinsic genetic or other characteristics, such as single-nucleotide polymorphisms, or preexisting diseases that result in an increase in internal dose, biologically effective dose, or target tissue response.

